# Association between multiple sclerosis and cancer risk: A two-sample Mendelian randomization study

**DOI:** 10.1371/journal.pone.0298271

**Published:** 2024-03-19

**Authors:** Zeyu Liu, Teng Fan, Xiaoyan Mo, Jun Kan, Bei Zhang

**Affiliations:** VIP Inpatient Department, State Key Laboratory of Oncology in South China, Guangdong Key Laboratory of Nasopharyngeal Carcinoma Diagnosis and Therapy, Sun Yat-sen University Cancer Center, Guangzhou, P. R. China; University of Newcastle, AUSTRALIA

## Abstract

Multiple Sclerosis (MS) is an immune-related disease and the relationship between MS and cancer has raised attention. Previous studies of the relationship between MS and cancer have reached conflicting conclusions. In this study, the two-sample MR method is used to investigate whether MS has a causal correlation with cancers and offer scientific evidence for cancer prevention. Single nucleotide polymorphisms (SNPs) related to MS were obtained from the genome-wide association study (GWAS) based on International Multiple Sclerosis Genetics Consortium (IMSGC) and SNPs related to 15 types of cancers were obtained from the GWASs based on UK Biobank. Inverse variance weighted (IVW) method was mainly used to assess causal effects. Sensitivity analyses were conducted with Cochran’s *Q*-test, MR Egger intercept, leave-one-out test, and MR Steiger method. IVW analysis showed that MS was only associated with a marginal increased risk of cervical cancer (OR 1.0004, 95% CI 1.0002–1.0007, *p* = 0.0003). Sensitivity analyses showed that the results of MR analysis were robust and found no heterogeneity, no pleiotropy, and no reverse causation. In conclusion, this study finds no causal relationship between MS and 15 types of cancers except cervical cancer.

## 1. Introduction

Multiple Sclerosis (MS) is a chronic central nervous system demyelinating disease, originating from complex gene-environment interactions and characterized by the accumulation of demyelinating lesions in the white matter and the grey matter of the brain and spinal cord [1]. Self-reactive lymph cell, largely CD4+ T cells, plays significant roles in the MS autoimmune inflammatory chaos [2]. The onset of MS is associated with both genetic and environmental factors. Familial MS cases constitute 12.6% of all MS patients, and the incidence of MS in monozygotic twins (up to 30% [3]) is significantly higher than that in dizygotic twins [4], underscoring the genetic predispositon. Recent research has identified multiple genetic loci related to MS susceptibility [5]. Meanwhile, environmental factors such as viral infections [6], smoking [7], and sunlight [8] may also have a role in the onset of MS.

Cancer remains a predominant global health concern, ranking as the second leading cause of morbidity and mortality, imposing a substantial disease burden [9]. Recently, significant progress has been made in cancer treatment, but the harm of cancer to human health and quality of life still remains a challenge [10, 11]. There are evidences that immune-related diseases are closely related to cancer [12], and the relationship between MS and cancer has also raised attention. Existing research has shown that earlier cancer detection is associated with reduced mortality for certain cancers [13]. Thus, it is critical to investigate whether MS patients possess an increased risk for cancer. Previous studies of the relationship between MS and cancer have reached conflicting conclusions. Some studies have shown that MS may reduce the risk of cancer [14], such as lung cancer [15] and alimentary tract cancers [16], while other studies have shown that MS is a risk factor for cancer, both in overall cancers [17] and in certain cancers such as bladder cancer [18], breast cancer [19], brain tumors [20], etc. Interestingly, a cohort study has found that after starting disease-modifying therapies for the treatment of MS in the 1990s, the risk of cancer increases in patients with MS [21], suggesting a potential relationship between MS immune suppressive treatment and cancer risk.

Mendelian randomization (MR) study is a useful epidemiological method to evaluate the hypothetical correlation between exposure and outcome [22]. Single nucleotide polymorphisms (SNPs) are used as instrumental variables (IVs) to assume the correlation in MR study. Given the autonomous segregation and random assignment of alleles at meiosis, MR can effectively mitigate potential confounders and reverse causation, thus providing a more reliable causal inference [23]. Hence, MR is a powerful tool to investigate the correction between MS and cancer. In this study, the two-sample MR method is used to assess the potential causal relationship between MS and various cancers and offer scientific evidence for cancer prevention.

## 2. Methods

### 2.1 Study design and data sources

**Fig 1:**
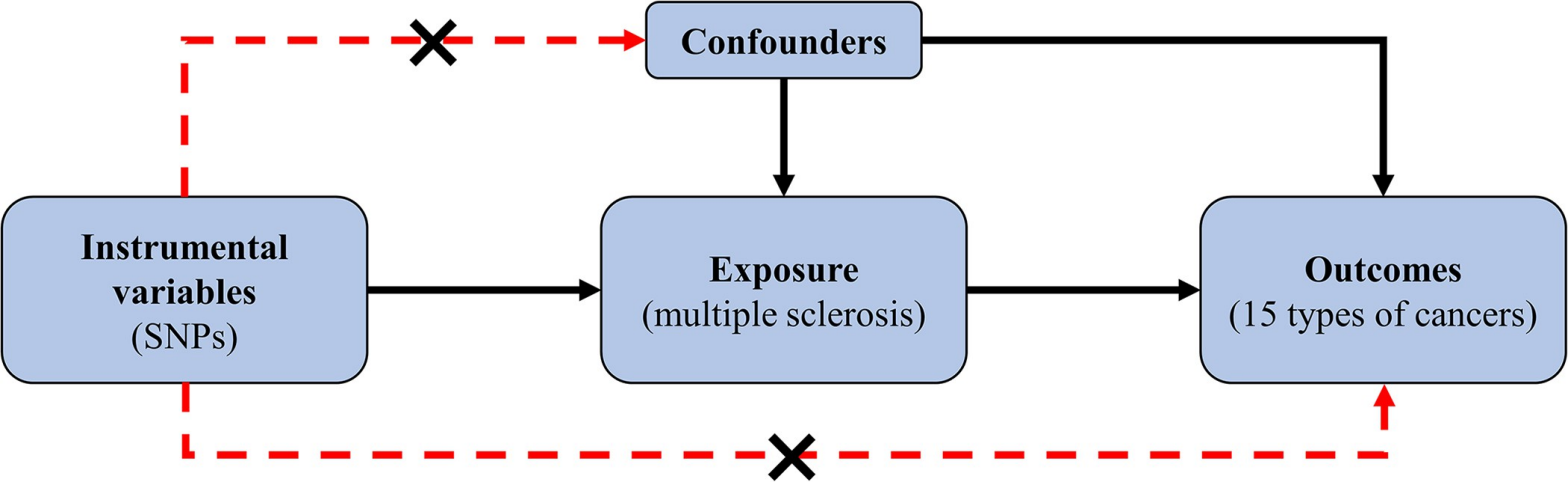
An overview of the Mendelian randomization study.

This study used the two-sample MR method to investigate the causal relationship between MS and cancer. The research flow chart was shown in Fig 1. The study is based on several hypotheses [24, 25]: first, genetic variation and MS are strongly correlated; second, genetic variation is independent of confounding factors; finally, genetic variants affect cancer risk only through MS and not through other ways.

The summary statistics of the GWAS used in this study were obtained from the Medical Research Council Integrative Epidemiology Unit OpenGWAS project (https://gwas.mrcieu.ac.uk/). Fifteen types of solid tumors (prostate cancer, breast cancer, bladder cancer, brain cancer, cervical cancer, laryngeal cancer, liver & bile duct cancer, lung cancer, malignant non-melanoma skin cancer, oesophageal cancer, oral cavity cancer, ovarian cancer, colorectal cancer, oropharyngeal cancer, melanoma skin cancer) were included as outcome traits, and these data were based on the UK Biobank consortium [26]. UK Biobank is a population-based prospective cohort which recruited more than 500000 UK volunteers aged 40 to 69 years and collected their genetic and health information [27]. The data of MS was based on International Multiple Sclerosis Genetics Consortium (IMSGC, https://imsgc.net/). IMSGC is an international collaborative organization dedicated to a comprehensively understanding of the genetic influences on etiology, pathogenesis, clinical course and treatment response of multiple sclerosis [28]. The summary of the data sources of exposure and outcome traits were shown in S1 Table. All data used in this study were obtained from publicly available databases; further ethical approval was not required.

### 2.2 Selection of instrumental variables

SNPs meeting the following criteria were selected as instrumental variables: (1) SNPs should be significantly correlated with multiple sclerosis (*p* < 1×10^−6^). (2) SNPs should be independent with each other to avoid the impact of linkage disequilibrium (LD), which means R^2^ < 0.001 and genetic distance kb > 10000. (3) SNPs should not be correlated with any confounders and outcomes. Phenoscanner database (http://www.phenoscanner.medschl.cam.ac.uk/) was used to identify associations with confounders and outcomes. We calculated *F*-statistic to evaluate the strength of the instruments and *F*-statistic > 10 indicated sufficient strength [29]. The calculation is: *F* = 2 × *MAF* × (1−*MAF*) *β*^2^ (*N*−2) / [1−2 × *MAF* × (1−*MAF*) *β*^2^], *MAF* is the minor allele frequency, *β* is the estimated corresponding effect and *N* is the exposure GWAS sample size.

### 2.3 Mendelian randomization analysis

Mendelian randomization analysis was performed by the TwoSampleMR package with R version 4.2.2. We mainly used inverse variance weighted (IVW) method to assess causal effects, but also referred to the results of four other methods (MR Egger, weighted median, simple mode, weighted mode). Results were expressed as odds ratios (OR) and 95% confidence intervals (CI). Bonferroni correction was performed on the *p* values of multiple testing. The significance threshold was set at 3.33×10^−3^ (0.05/15). In sensitivity analysis, we used Cochran’s *Q*-test to assess heterogeneity, MR Egger intercept for pleiotropy [30], leave-one-out test to assess robustness of results, and MR Steiger method to conduct the directionality test [31].

## 3. Results

We screened out 107 SNPs from the GWAS data of the exposure factors, all of which had a strong correlation with MS (*p* < 1×10^−6^), and the 107 SNPs were independent of each other without LD (S2 Table). Upon a review of the literature related to MS and cancer, we identified several potential confounders. Evidence suggests that smoking is a risk factor for MS [7], with risks increasing with cumulative smoking exposure [32]. Prolonged exposure to tobacco can accelerate the progression of MS [33]. Smoking is also a recognized cancer risk factor [34]. Epstein-Barr virus infection significantly elevates the risk for MS [6] and is also a risk factor for several cancers [35], such as nasopharyngeal carcinoma [36], gastric cancer [37], and lymphomas [38]. We searched the Phenoscanner database for phenotypes associated with the screened SNPs and found 5 confounding factors that might be associated with MS and cancer. The 102 SNPs were normalized to the outcome data to obtain the final IVs. All IVs had *F*-statistics > 10, indicating that they were not weak instruments.

**Fig 2:**
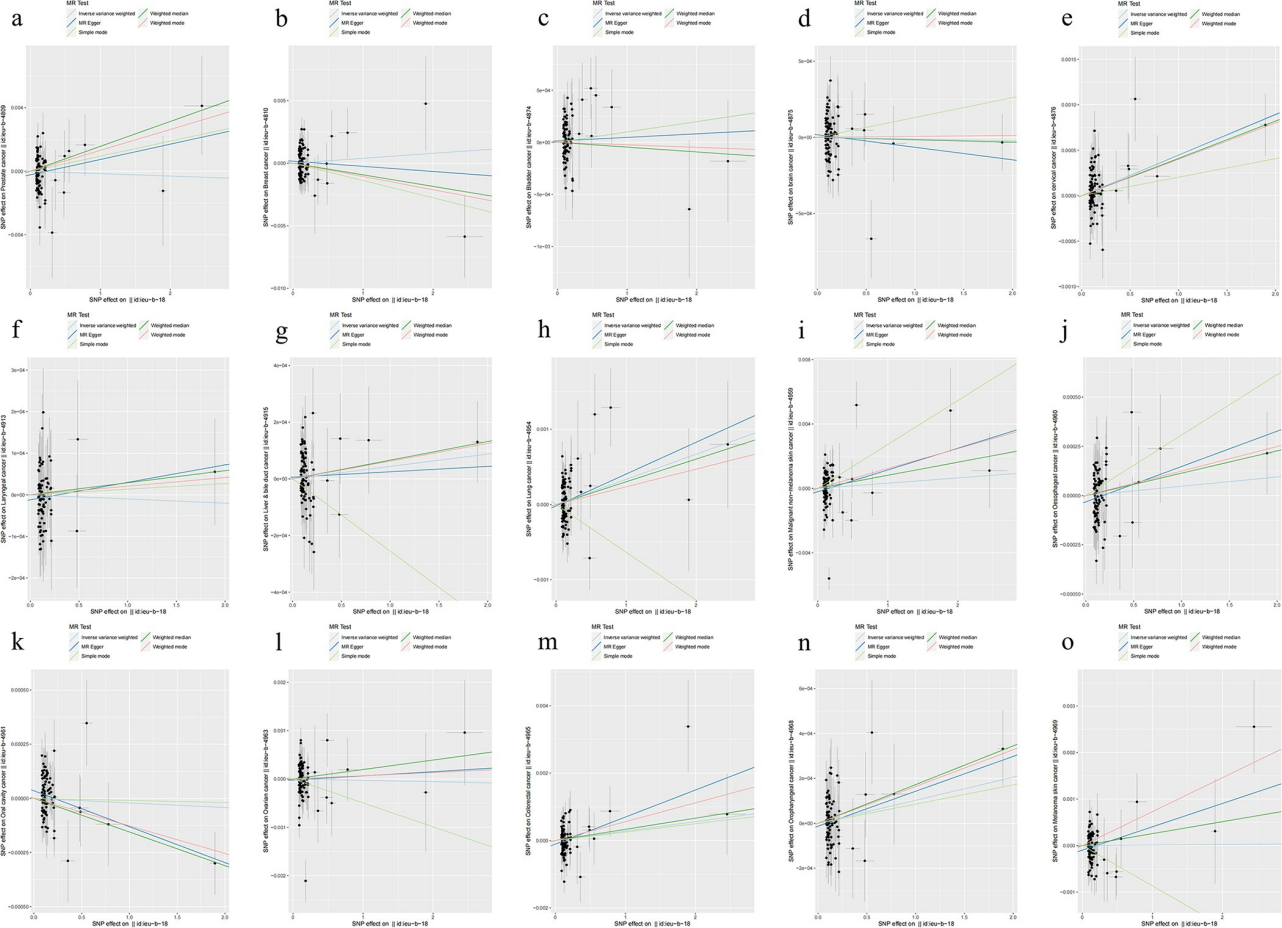
Scatter plots of the MR results: (a) prostate cancer, (b) breast cancer, (c) bladder cancer, (d) brain cancer, (e) cervical cancer, (f) laryngeal cancer, (g) liver & bile duct cancer, (h) lung cancer, (i) malignant non-melanoma skin cancer, (j) oesophageal cancer, (k) oral cavity cancer, (l) ovarian cancer, (m) colorectal cancer, (n) oropharyngeal cancer, (o) melanoma skin cancer.

The results of the causal analysis of MS and 15 types of cancers were shown in the Table 1. The determination of causality mainly referred to the results of IVW analysis. In general, the MR results showed no causal relationship between MS and almost all cancers. IVW analysis showed that MS was only associated with higher risk of cervical cancer (OR 1.0004, 95% CI 1.0002–1.0007, *p* = 0.0003). After multiple testing correction, the *p* value of cervical cancer was still less than 3.33×10^−3^ (0.05/15), indicating that MS was a risk factor for cervical cancer. Scatter plots of the MR results were shown in the Fig 2.

**Table 1:** Causal analysis of MS and 15 types of cancers.

Outcome	Method	SNP	OR (95% CI)	*p* value
**Prostate cancer**	MR Egger	92	1.0010(0.9990, 1.0029)	0.3357
	Weighted median	92	1.0016(0.9999, 1.0032)	0.0652
	Inverse variance weighted	92	0.9998(0.9986, 1.0011)	0.8037
	Simple mode	92	1.0009(0.9971, 1.0048)	0.6310
	Weighted mode	92	1.0013(0.9995, 1.0032)	0.1652
**Breast cancer**	MR Egger	92	0.9996(0.9975, 1.0017)	0.7057
	Weighted median	92	0.9991(0.9972, 1.0010)	0.3348
	Inverse variance weighted	92	1.0004(0.9991, 1.0017)	0.5484
	Simple mode	92	0.9986(0.9945, 1.0027)	0.5125
	Weighted mode	92	0.9989(0.9968, 1.0011)	0.3426
**Bladder cancer**	MR Egger	92	1.0000(0.9997, 1.0004)	0.8530
	Weighted median	92	1.0000(0.9996, 1.0003)	0.8011
	Inverse variance weighted	92	1.0001(0.9999, 1.0003)	0.3744
	Simple mode	92	1.0001(0.9994, 1.0008)	0.7807
	Weighted mode	92	1.0000(0.9996, 1.0003)	0.8985
**brain cancer**	MR Egger	90	0.9999(0.9997, 1.0001)	0.4464
	Weighted median	90	1.0000(0.9998, 1.0002)	0.8707
	Inverse variance weighted	90	1.0000(0.9998, 1.0001)	0.8739
	Simple mode	90	1.0001(0.9996, 1.0006)	0.6176
	Weighted mode	90	1.0000(0.9998, 1.0002)	0.9492
**cervical cancer**	MR Egger	90	1.0004(1.0001, 1.0008)	0.0141
	Weighted median	90	1.0004(1.0001, 1.0008)	0.0202
	Inverse variance weighted	90	1.0004(1.0002, 1.0007)	0.0003
	Simple mode	90	1.0002(0.9994, 1.0010)	0.6203
	Weighted mode	90	1.0004(1.0001, 1.0007)	0.0202
**Laryngeal cancer**	MR Egger	87	1.0000(0.9999, 1.0002)	0.5282
	Weighted median	87	1.0000(0.9999, 1.0002)	0.6518
	Inverse variance weighted	87	1.0000(0.9999, 1.0001)	0.8215
	Simple mode	87	1.0000(0.9997, 1.0004)	0.9377
	Weighted mode	87	1.0000(0.9999, 1.0001)	0.7312
**Liver & bile duct cancer**	MR Egger	89	1.0000(0.9999, 1.0002)	0.7995
	Weighted median	89	1.0001(0.9999, 1.0002)	0.3577
	Inverse variance weighted	89	1.0000(0.9999, 1.0001)	0.3864
	Simple mode	89	0.9997(0.9993, 1.0001)	0.2104
	Weighted mode	89	1.0001(0.9999, 1.0002)	0.3412
**Lung cancer**	MR Egger	92	1.0004(0.9999, 1.0010)	0.1352
	Weighted median	92	1.0003(0.9997, 1.0009)	0.2956
	Inverse variance weighted	92	1.0003(1.0000, 1.0007)	0.0498
	Simple mode	92	0.9994(0.9980, 1.0007)	0.3533
	Weighted mode	92	1.0002(0.9997, 1.0008)	0.4329
**Malignant non-melanoma skin cancer**	MR Egger	92	1.0014(0.9992, 1.0035)	0.2245
	Weighted median	92	1.0008(0.9993, 1.0024)	0.3020
	Inverse variance weighted	92	1.0003(0.9990, 1.0016)	0.6314
	Simple mode	92	1.0027(0.9994, 1.0060)	0.1081
	Weighted mode	92	1.0012(0.9997, 1.0027)	0.1099
**Oesophageal cancer**	MR Egger	90	1.0002(1.0000, 1.0004)	0.0879
	Weighted median	90	1.0001(0.9999, 1.0003)	0.2945
	Inverse variance weighted	90	1.0000(0.9999, 1.0002)	0.5105
	Simple mode	90	1.0003(0.9998, 1.0008)	0.2556
	Weighted mode	90	1.0001(0.9999, 1.0003)	0.2578
**Oral cavity cancer**	MR Egger	90	0.9998(0.9997, 1.0000)	0.0270
	Weighted median	90	0.9998(0.9997, 1.0000)	0.0373
	Inverse variance weighted	90	1.0000(0.9999, 1.0001)	0.6826
	Simple mode	90	1.0000(0.9996, 1.0004)	0.9636
	Weighted mode	90	0.9999(0.9997, 1.0000)	0.0792
**Ovarian cancer**	MR Egger	92	1.0001(0.9994, 1.0008)	0.8158
	Weighted median	92	1.0002(0.9996, 1.0008)	0.5159
	Inverse variance weighted	92	1.0000(0.9995, 1.0004)	0.9004
	Simple mode	92	0.9995(0.9983, 1.0007)	0.4159
	Weighted mode	92	1.0001(0.9995, 1.0007)	0.8308
**Colorectal cancer**	MR Egger	92	1.0008(1.0000, 1.0016)	0.0547
	Weighted median	92	1.0003(0.9995, 1.0011)	0.4108
	Inverse variance weighted	92	1.0003(0.9998, 1.0008)	0.2711
	Simple mode	92	1.0002(0.9987, 1.0018)	0.7564
	Weighted mode	92	1.0006(0.9998, 1.0013)	0.1485
**Oropharyngeal cancer**	MR Egger	90	1.0002(1.0000, 1.0003)	0.0712
	Weighted median	90	1.0002(1.0000, 1.0003)	0.0510
	Inverse variance weighted	90	1.0001(1.0000, 1.0002)	0.0772
	Simple mode	90	1.0001(0.9996, 1.0005)	0.7052
	Weighted mode	90	1.0002(1.0000, 1.0003)	0.0461
**Melanoma skin cancer**	MR Egger	92	1.0005(0.9999, 1.0011)	0.1248
	Weighted median	92	1.0003(0.9997, 1.0009)	0.3979
	Inverse variance weighted	92	1.0000(0.9996, 1.0004)	0.9544
	Simple mode	92	0.9991(0.9978, 1.0005)	0.2228
	Weighted mode	92	1.0007(1.0001, 1.0013)	0.0216

**Fig 3:**
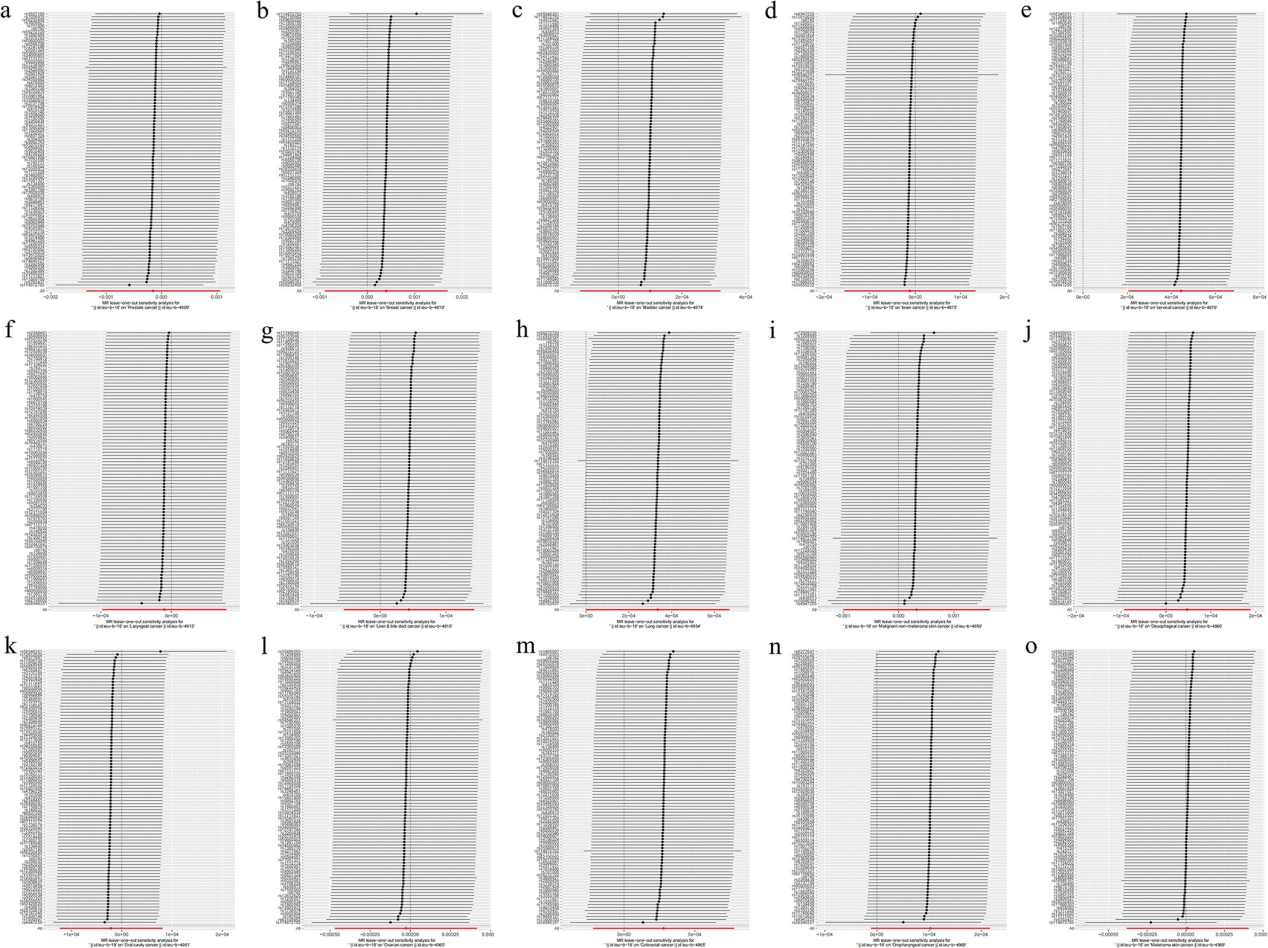
Leave-one-out test results: (a) prostate cancer, (b) breast cancer, (c) bladder cancer, (d) brain cancer, (e) cervical cancer, (f) laryngeal cancer, (g) liver & bile duct cancer, (h) lung cancer, (i) malignant non-melanoma skin cancer, (j) oesophageal cancer, (k) oral cavity cancer, (l) ovarian cancer, (m) colorectal cancer, (n) oropharyngeal cancer, (o) melanoma skin cancer.

Sensitivity analyses were conducted to verify the reliability of MR results. The Cochran’s *Q*-test found no heterogeneity in cervical cancer (Table 2), and the MR Egger intercept method found no pleiotropy in cervical cancer (Table 3). The MR Steiger directionality test found no reverse causation (Table 4), and the leave-one-out test showed that the results of MR analysis were robust (Fig 3).

**Table 2:** Cochran’s Q-test.

Outcome	Method	*Q*	*Q*_df	*Q*_*p*val
**Prostate cancer**	MR Egger	111.2732	90	0.0637
	Inverse variance weighted	113.7652	91	0.0534
**Breast cancer**	MR Egger	116.6085	90	0.0311
	Inverse variance weighted	117.7485	91	0.0311
**Bladder cancer**	MR Egger	106.7965	90	0.1092
	Inverse variance weighted	107.0348	91	0.1202
**brain cancer**	MR Egger	115.0544	88	0.0280
	Inverse variance weighted	116.1095	89	0.0284
**cervical cancer**	MR Egger	98.0164	88	0.2183
	Inverse variance weighted	98.0173	89	0.2407
**Laryngeal cancer**	MR Egger	55.6793	85	0.9942
	Inverse variance weighted	56.8075	86	0.9936
**Liver & bile duct cancer**	MR Egger	92.0023	87	0.3363
	Inverse variance weighted	92.2195	88	0.3582
**Lung cancer**	MR Egger	119.6247	90	0.0201
	Inverse variance weighted	119.8344	91	0.0231
**Malignant non-melanoma skin cancer**	MR Egger	246.0540	90	0.0000
	Inverse variance weighted	249.8197	91	0.0000
**Oesophageal cancer**	MR Egger	88.0616	88	0.4781
	Inverse variance weighted	91.0891	89	0.4187
**Oral cavity cancer**	MR Egger	91.4637	88	0.3791
	Inverse variance weighted	98.9387	89	0.2211
**Ovarian cancer**	MR Egger	128.8383	90	0.0046
	Inverse variance weighted	129.0560	91	0.0054
**Colorectal cancer**	MR Egger	124.5669	90	0.0093
	Inverse variance weighted	128.1216	91	0.0063
**Oropharyngeal cancer**	MR Egger	85.1771	88	0.5654
	Inverse variance weighted	85.8976	89	0.5734
**Melanoma skin cancer**	MR Egger	114.4810	90	0.0418
	Inverse variance weighted	119.1332	91	0.0255

**Table 3:** MR Egger intercept.

Outcome	Egger_intercept	SE	pval
**Prostate cancer**	-2.25E-04	1.59E-04	0.1591
**Breast cancer**	1.61E-04	1.72E-04	0.3507
**Bladder cancer**	1.31E-05	2.93E-05	0.6552
**brain cancer**	1.71E-05	1.90E-05	0.3714
**cervical cancer**	-9.20E-07	3.16E-05	0.9768
**Laryngeal cancer**	-1.21E-05	1.14E-05	0.2912
**Liver & bile duct cancer**	5.91E-06	1.31E-05	0.6516
**Lung cancer**	-1.77E-05	4.46E-05	0.6921
**Malignant non-melanoma skin cancer**	-2.06E-04	1.75E-04	0.2436
**Oesophageal cancer**	-3.19E-05	1.84E-05	0.0855
**Oral cavity cancer**	3.49E-05	1.30E-05	0.0087
**Ovarian cancer**	-2.30E-05	5.89E-05	0.6974
**Colorectal cancer**	-1.05E-04	6.55E-05	0.1125
**Oropharyngeal cancer**	-1.27E-05	1.50E-05	0.3983
**Melanoma skin cancer**	-9.85E-05	5.15E-05	0.0590

**Table 4:** MR Steiger directionality test.

Outcome	Snp_r^2^ (exposure)	Snp_r^2^ (outcome)	Correct causal direction	Steiger *p*val
**Prostate cancer**	0.0453	0.0008	TRUE	0
**Breast cancer**	0.0453	0.0006	TRUE	0
**Bladder cancer**	0.0453	0.0003	TRUE	0
**brain cancer**	0.0443	0.0003	TRUE	0
**cervical cancer**	0.0443	0.0006	TRUE	0
**Laryngeal cancer**	0.0429	0.0002	TRUE	0
**Liver & bile duct cancer**	0.0434	0.0003	TRUE	0
**Lung cancer**	0.0453	0.0004	TRUE	0
**Malignant non-melanoma skin cancer**	0.0453	0.0008	TRUE	0
**Oesophageal cancer**	0.0443	0.0003	TRUE	0
**Oral cavity cancer**	0.0443	0.0003	TRUE	0
**Ovarian cancer**	0.0453	0.0007	TRUE	0
**Colorectal cancer**	0.0453	0.0004	TRUE	0
**Oropharyngeal cancer**	0.0443	0.0003	TRUE	0
**Melanoma skin cancer**	0.0453	0.0003	TRUE	0

## 4. Discussions

In this study, we evaluated the causal relationship between MS and 15 types of cancers. Our findings indicate that genetic susceptibility to MS is associated with an increased risk of cervical cancer and no other types of cancer. Sensitivity analyses confirmed that these outcomes were robust.

MR employs genetic variations as IVs to assess causality between the exposure and the outcome. Since alleles are randomly assigned to offspring, MR can effectively avoid reverse causation. A previous study used MR to analyze the relationship between MS and lung cancer [39], and found that MS increased the risk of overall lung cancer but there were no significant causal relationships between MS and lung adenocarcinoma or squamous cell cancer. By contrast, no causal association between MS and lung cancer risk was found in our study. The difference was possibly due to heterogeneity in the GWAS population included in the two studies. Another study explored the association between MS and breast cancer based on MR analysis and the result does not support the correlation between them [40], which is consistent with our results.

We found that MS was associated with a marginal increased risk of cervical cancer through MR and remained significant after multiple testing correction. A previous cohort study identified that cervical benign cellular changes, considered as a premalignant condition, was more frequently observed in MS patients treated with cytotoxic immunosuppressive agents [41]. A clinical trial on the safety and tolerability of cladribine tablets in multiple sclerosis reported pre-malignant cervical carcinoma in situ [42]. Additionally, there are several case reports of patients developing cervical dysplasia during natalizumab therapy [43], and in some instances, rapidly progressing to squamous cell carcinoma of the cervix [44]. Researchers suspect that the occurrence of premalignant conditions and cervical cancer might be associated with the drugs used in MS treatment. The pathogenesis of MS involves disrupted immune function; hence, most current therapies, including corticosteroids and disease-modifying therapies (DMTs), have immunosuppressive effects. Some agents with immunosuppressive effect (i.e. S1P receptor modulators) might reduce the number of lymphocytes that are needed to identify and eliminate malignant cells [45]. The immunosuppression may also hinder the body’s ability to clear HPV [46], a well-known risk factor for cervical cancer. Besides, medications for treating MS could promote extramedullary hematopoiesis and myeloid-derived suppressor cells accumulation, thus it might have a protumorigenic potential [47]. However, it’s crucial to note that, according to our analysis of publicly available GWAS data, MS is associated with only a slight increase (0.04%) in cervical cancer risk. Due to the limitations of data sources and sample size, obtaining a more precise odds ratio is challenging and necessitates further epidemiological studies. Although the identified increase in risk is minimal, the growing body of evidence suggesting a potential rise in cervical cancer risk due to MS medications underscores the need for vigilant post-treatment monitoring, including routine cervical HPV testing, histological examination, and HPV vaccination for MS patients.

Compared with conventional epidemiological studies, our study’s strength lies in its application of MR to discern the causal relationship between MS and cancer. By using genetic variations as the IVs, we minimized the impacts of confounders and reverse causation. In this study, there is small sample overlap, ensuring the independence of exposure and outcomes. Different methods are used for sensitivity analyses to find out potential pleiotropy and heterogeneity, making MR results more robust. Compared to previous MR studies on MS and cancer, this study covers a wider range of cancer types.

However, our study also has some limitations. First, the populations included were exclusively of European ancestry, limiting its generalizability to other ethnicities. Second, although we excluded potential confounding factors and the influence of gene pleiotropy on the results as much as possible, residual effects might exist. Third, drug use in MS patients is not recorded and a deeper analysis of the effects of drugs is not possible. Fourth, the increased risk of cervical cancer is marginal and more studies are required to confirm this finding. Lastly, while MR analysis offers a preliminary insight into the MS-cancer causality, the intricate biological mechanisms underpinning this relationship remain elusive and warrant further exploration in laboratory settings.

## 5. Conclusion

In conclusion, by using MR method, this study reveals that MS is only causally associated with a marginal increased risk of cervical cancer and shows no association with other types of cancer. The specific biological mechanism remains to be studied.

## Supporting information

S1 Table Information on exposure and outcome data sources. (DOCX)

S2 Table Detailed statistics of selected instrumental variables for multiple sclerosis. (DOCX)

## Abbreviations

MS: multiple sclerosis
MR: Mendelian randomization
SNP: single nucleotide polymorphism
IV: instrumental variable
LD: linkage disequilibrium
GWAS: genome-wide association study
IVW: inverse variance weight
OR: odds ratio
CI: confidence interval
DMT: disease-modifying therapy
