# Supplementary material for: Association between multiple sclerosis and cancer risk: A two-sample Mendelian randomization study
Source: PLoS One. 2024 Mar 19;19(3):e0298271. doi: 10.1371/journal.pone.0298271 (PMC10950213; doi:10.1371/journal.pone.0298271)
Supplement: S1 Table — (DOCX) [file pone.0298271.s001.docx]

| **Trait** | **GWAS ID** | **Consortium** | **Sample size** |
| --- | --- | --- | --- |
| Multiple sclerosis | ieu-b-18 | IMSGC | 115,803 |
| Prostate cancer | ieu-b-4809 | UK Biobank | 182,625 |
| Breast cancer | ieu-b-4810 | UK Biobank | 212,402 |
| Bladder cancer | ieu-b-4874 | UK Biobank | 373,295 |
| Brain cancer | ieu-b-4875 | UK Biobank | 372,622 |
| Cervical cancer | ieu-b-4876 | UK Biobank | 199,086 |
| Laryngeal cancer | ieu-b-4913 | UK Biobank | 372,289 |
| Liver & bile duct cancer | ieu-b-4915 | UK Biobank | 372,366 |
| Lung cancer | ieu-b-4954 | UK Biobank | 374,687 |
| Malignant non-melanoma skin cancer | ieu-b-4959 | UK Biobank | 395,710 |
| Oesophageal cancer | ieu-b-4960 | UK Biobank | 372,756 |
| Oral cavity cancer | ieu-b-4961 | UK Biobank | 372,373 |
| Ovarian cancer | ieu-b-4963 | UK Biobank | 199,741 |
| Colorectal cancer | ieu-b-4965 | UK Biobank | 377,673 |
| Oropharyngeal cancer | ieu-b-4968 | UK Biobank | 372,510 |
| Melanoma skin cancer | ieu-b-4969 | UK Biobank | 375,767 |

S1 Table Information on exposure and outcome data sources.
