# Supplementary material for: Association between multiple sclerosis and cancer risk: A two-sample Mendelian randomization study
Source: PLoS One. 2024 Mar 19;19(3):e0298271. doi: 10.1371/journal.pone.0298271 (PMC10950213; doi:10.1371/journal.pone.0298271)
Supplement: S2 Table — (DOCX) [file pone.0298271.s002.docx]

| SNP | chromosome | position | effect allele | β | se | p | R^2^ | F |
| --- | --- | --- | --- | --- | --- | --- | --- | --- |
| rs10801908 | 1 | 117090493 | T | -0.21 | 0.03 | 3.54E-16 | 5.74E-04 | 66.48 |
| rs59655222 | 1 | 200875897 | C | -0.12 | 0.02 | 3.76E-11 | 3.78E-04 | 43.74 |
| rs478093 | 1 | 120255126 | G | 0.11 | 0.02 | 4.30E-09 | 2.98E-04 | 34.48 |
| rs3737798 | 1 | 160389984 | G | -0.09 | 0.02 | 1.40E-07 | 2.39E-04 | 27.72 |
| rs74449127 | 1 | 101290432 | G | -0.20 | 0.03 | 1.36E-14 | 5.12E-04 | 59.29 |
| rs10914539 | 1 | 32715641 | T | 0.13 | 0.03 | 5.35E-07 | 2.17E-04 | 25.13 |
| rs6670198 | 1 | 2520527 | C | -0.15 | 0.02 | 2.03E-16 | 5.83E-04 | 67.57 |
| rs2317231 | 1 | 157686337 | T | -0.10 | 0.02 | 1.90E-09 | 3.11E-04 | 36.07 |
| rs35486093 | 1 | 85729820 | G | 0.18 | 0.03 | 1.60E-10 | 3.53E-04 | 40.90 |
| rs11809700 | 1 | 93152635 | T | 0.14 | 0.02 | 3.51E-15 | 5.35E-04 | 61.96 |
| rs62195662 | 2 | 209561980 | C | 0.49 | 0.09 | 2.47E-07 | 2.30E-04 | 26.62 |
| rs35540610 | 2 | 231121829 | C | 0.14 | 0.02 | 2.88E-12 | 4.21E-04 | 48.77 |
| rs1177228 | 2 | 61242410 | G | 0.11 | 0.02 | 8.57E-09 | 2.86E-04 | 33.14 |
| rs7592560 | 2 | 68647001 | A | 0.10 | 0.02 | 2.87E-10 | 3.43E-04 | 39.76 |
| rs12478539 | 2 | 43355324 | C | -0.12 | 0.02 | 4.37E-11 | 3.75E-04 | 43.44 |
| rs12612620 | 2 | 112488876 | A | 0.21 | 0.04 | 4.26E-09 | 2.98E-04 | 34.50 |
| rs1014486 | 3 | 159691112 | C | 0.11 | 0.02 | 1.36E-10 | 3.56E-04 | 41.21 |
| rs6763437 | 3 | 119145390 | A | -0.78 | 0.13 | 3.72E-09 | 3.00E-04 | 34.76 |
| rs11711621 | 3 | 169524016 | T | -0.10 | 0.02 | 5.63E-08 | 2.55E-04 | 29.49 |
| rs2681424 | 3 | 121769522 | C | -0.12 | 0.02 | 2.71E-13 | 4.61E-04 | 53.41 |
| rs13327021 | 3 | 27783015 | T | 0.12 | 0.02 | 1.73E-11 | 3.91E-04 | 45.26 |
| rs9878602 | 3 | 71535338 | G | -0.08 | 0.02 | 2.60E-07 | 2.29E-04 | 26.53 |
| rs4325907 | 3 | 101749022 | T | -0.10 | 0.02 | 3.68E-09 | 3.00E-04 | 34.79 |
| rs438613 | 3 | 28072086 | C | 0.14 | 0.02 | 9.43E-17 | 5.96E-04 | 69.08 |
| rs6837324 | 4 | 48127262 | G | 0.09 | 0.02 | 3.16E-07 | 2.26E-04 | 26.15 |
| rs9992763 | 4 | 109058718 | T | -0.09 | 0.02 | 4.51E-08 | 2.58E-04 | 29.92 |
| rs2705616 | 4 | 87862396 | G | -0.08 | 0.02 | 4.35E-07 | 2.20E-04 | 25.53 |
| rs17051321 | 4 | 122119449 | T | 0.09 | 0.02 | 4.95E-07 | 2.18E-04 | 25.28 |
| rs67111717 | 5 | 176790162 | G | 0.10 | 0.02 | 4.96E-07 | 2.18E-04 | 25.28 |
| rs10063294 | 5 | 35877505 | A | -0.10 | 0.02 | 1.13E-09 | 3.20E-04 | 37.09 |
| rs11749040 | 5 | 40396425 | A | 0.20 | 0.02 | 3.54E-17 | 6.13E-04 | 71.02 |
| rs9327104 | 5 | 118883648 | G | 0.31 | 0.06 | 4.41E-07 | 2.20E-04 | 25.50 |
| rs2546890 | 5 | 158759900 | G | -0.12 | 0.02 | 1.04E-12 | 4.38E-04 | 50.77 |
| rs9393975 | 6 | 29802217 | A | -0.77 | 0.04 | 1.49E-70 | 2.72E-03 | 315.35 |
| rs743771 | 6 | 32976909 | A | -0.10 | 0.02 | 8.55E-10 | 3.25E-04 | 37.63 |
| rs72928038 | 6 | 90976768 | A | 0.16 | 0.02 | 9.01E-11 | 3.63E-04 | 42.03 |
| rs2857700 | 6 | 31572481 | C | -0.77 | 0.02 | 1.00E-200 | 8.93E-03 | 1043.60 |
| rs9277647 | 6 | 33083750 | T | -0.21 | 0.02 | 5.47E-24 | 8.80E-04 | 102.03 |
| rs1738074 | 6 | 159465977 | C | 0.11 | 0.02 | 9.91E-12 | 4.00E-04 | 46.35 |
| rs56272720 | 6 | 135532832 | G | 0.48 | 0.09 | 1.63E-07 | 2.37E-04 | 27.43 |
| rs4896153 | 6 | 135833463 | A | -0.14 | 0.02 | 1.65E-13 | 4.69E-04 | 54.39 |
| rs4947255 | 6 | 32207483 | T | -0.55 | 0.06 | 2.54E-23 | 8.54E-04 | 98.99 |
| rs114872782 | 6 | 32241452 | T | -2.45 | 0.26 | 7.72E-22 | 7.96E-04 | 92.23 |
| rs16822584 | 6 | 32407537 | T | -0.29 | 0.06 | 1.60E-07 | 2.37E-04 | 27.47 |
| rs62420820 | 6 | 137438057 | A | 0.14 | 0.02 | 2.50E-13 | 4.62E-04 | 53.57 |
| rs58546351 | 6 | 32483611 | G | -1.89 | 0.05 | 1.00E-200 | 1.08E-02 | 1269.24 |
| rs12211604 | 6 | 7100029 | A | -0.09 | 0.02 | 1.86E-07 | 2.35E-04 | 27.17 |
| rs1997768 | 6 | 26217728 | G | -0.12 | 0.02 | 9.06E-07 | 2.08E-04 | 24.12 |
| rs60600003 | 7 | 37382465 | G | 0.13 | 0.03 | 4.20E-07 | 2.21E-04 | 25.60 |
| rs2242508 | 7 | 56151489 | G | -0.09 | 0.02 | 2.94E-07 | 2.27E-04 | 26.29 |
| rs354033 | 7 | 149289464 | A | -0.11 | 0.02 | 1.21E-08 | 2.80E-04 | 32.48 |
| rs55970742 | 7 | 2441337 | T | -0.10 | 0.02 | 2.05E-08 | 2.71E-04 | 31.45 |
| rs7385730 | 7 | 50318938 | T | -0.12 | 0.02 | 3.99E-07 | 2.22E-04 | 25.70 |
| rs6990534 | 8 | 128814091 | G | 0.11 | 0.02 | 3.60E-09 | 3.01E-04 | 34.83 |
| rs7830997 | 8 | 71442114 | G | -0.08 | 0.02 | 9.87E-07 | 2.07E-04 | 23.95 |
| rs28703878 | 8 | 79417222 | G | 0.13 | 0.02 | 4.51E-10 | 3.36E-04 | 38.88 |
| rs7855251 | 9 | 100868189 | C | -0.11 | 0.02 | 4.23E-08 | 2.59E-04 | 30.04 |
| rs1250551 | 10 | 81059335 | T | 0.12 | 0.02 | 2.66E-11 | 3.83E-04 | 44.41 |
| rs1112718 | 10 | 94479107 | G | -0.11 | 0.02 | 2.46E-10 | 3.46E-04 | 40.06 |
| rs11256593 | 10 | 6117322 | T | 0.19 | 0.02 | 6.78E-27 | 9.95E-04 | 115.30 |
| rs61884005 | 11 | 14402930 | G | -0.13 | 0.03 | 8.02E-08 | 2.49E-04 | 28.80 |
| rs2269434 | 11 | 47360412 | C | 0.09 | 0.02 | 5.24E-07 | 2.17E-04 | 25.17 |
| rs56232455 | 11 | 321235 | A | 0.16 | 0.03 | 1.78E-08 | 2.74E-04 | 31.72 |
| rs12365699 | 11 | 118743286 | A | -0.14 | 0.02 | 3.15E-10 | 3.42E-04 | 39.58 |
| rs6589706 | 11 | 118747813 | G | -0.08 | 0.02 | 5.88E-07 | 2.15E-04 | 24.95 |
| rs4939490 | 11 | 60793651 | G | 0.14 | 0.02 | 4.25E-15 | 5.32E-04 | 61.58 |
| rs1204649 | 11 | 65702776 | C | -0.09 | 0.02 | 1.24E-07 | 2.41E-04 | 27.95 |
| rs701006 | 12 | 58106836 | G | 0.11 | 0.02 | 1.35E-11 | 3.95E-04 | 45.74 |
| rs7975763 | 12 | 123604053 | T | 0.12 | 0.02 | 7.80E-09 | 2.88E-04 | 33.32 |
| rs1860545 | 12 | 6446777 | A | 0.12 | 0.02 | 7.79E-12 | 4.04E-04 | 46.82 |
| rs3783196 | 13 | 42039288 | A | -0.08 | 0.02 | 4.34E-07 | 2.20E-04 | 25.54 |
| rs9591325 | 13 | 50811220 | C | -0.21 | 0.03 | 4.16E-10 | 3.37E-04 | 39.04 |
| rs9557185 | 13 | 99895107 | T | -0.13 | 0.03 | 5.97E-07 | 2.15E-04 | 24.92 |
| rs11624343 | 14 | 103859156 | A | 0.08 | 0.02 | 7.33E-07 | 2.12E-04 | 24.53 |
| rs17124032 | 14 | 88546009 | A | -0.22 | 0.03 | 7.08E-12 | 4.06E-04 | 47.00 |
| rs12147246 | 14 | 103265844 | G | -0.10 | 0.02 | 4.29E-09 | 2.98E-04 | 34.49 |
| rs34695601 | 14 | 76014298 | C | -0.11 | 0.02 | 3.16E-08 | 2.64E-04 | 30.60 |
| rs12434551 | 14 | 69253364 | T | -0.10 | 0.02 | 1.83E-10 | 3.51E-04 | 40.64 |
| rs6496663 | 15 | 90887584 | C | 0.10 | 0.02 | 2.78E-08 | 2.66E-04 | 30.86 |
| rs11629628 | 15 | 89261248 | A | 0.36 | 0.07 | 5.07E-07 | 2.18E-04 | 25.24 |
| rs34550882 | 16 | 31274875 | T | 0.14 | 0.03 | 6.51E-07 | 2.14E-04 | 24.76 |
| rs35703946 | 16 | 86021505 | A | -0.17 | 0.03 | 1.94E-09 | 3.11E-04 | 36.04 |
| rs3809627 | 16 | 30103160 | A | -0.10 | 0.02 | 3.25E-08 | 2.64E-04 | 30.55 |
| rs17724508 | 16 | 79350204 | C | -0.21 | 0.04 | 5.30E-08 | 2.56E-04 | 29.61 |
| rs7190580 | 16 | 11403470 | G | -0.10 | 0.02 | 4.64E-08 | 2.58E-04 | 29.86 |
| rs6564681 | 16 | 79652720 | T | -0.09 | 0.02 | 1.80E-07 | 2.35E-04 | 27.24 |
| rs415759 | 16 | 1066917 | C | 0.12 | 0.02 | 4.76E-08 | 2.57E-04 | 29.81 |
| rs7200146 | 16 | 11213449 | T | -0.17 | 0.02 | 7.00E-24 | 8.76E-04 | 101.54 |
| rs8062446 | 16 | 57077094 | T | 0.08 | 0.02 | 8.41E-07 | 2.09E-04 | 24.26 |
| rs12925972 | 16 | 79111297 | C | 0.09 | 0.02 | 3.07E-08 | 2.65E-04 | 30.66 |
| rs4796224 | 17 | 34842521 | G | 0.09 | 0.02 | 1.62E-07 | 2.37E-04 | 27.44 |
| rs7207542 | 17 | 45697549 | G | 0.11 | 0.02 | 4.67E-11 | 3.74E-04 | 43.31 |
| rs1026916 | 17 | 40529835 | G | -0.13 | 0.02 | 1.02E-13 | 4.78E-04 | 55.33 |
| rs2150879 | 17 | 57859210 | A | -0.10 | 0.02 | 3.29E-10 | 3.41E-04 | 39.49 |
| rs9955954 | 18 | 56348044 | G | -0.11 | 0.02 | 1.54E-08 | 2.76E-04 | 32.00 |
| rs1077667 | 19 | 6668972 | T | -0.15 | 0.02 | 8.37E-13 | 4.42E-04 | 51.19 |
| rs11666263 | 19 | 10590684 | G | -0.10 | 0.02 | 1.20E-08 | 2.81E-04 | 32.49 |
| rs6509314 | 19 | 47696626 | T | 0.10 | 0.02 | 5.39E-07 | 2.17E-04 | 25.12 |
| rs4808760 | 19 | 18301979 | G | -0.13 | 0.02 | 4.84E-13 | 4.51E-04 | 52.27 |
| rs1465697 | 19 | 49837246 | T | 0.12 | 0.02 | 3.48E-11 | 3.79E-04 | 43.89 |
| rs6032662 | 20 | 44734310 | T | -0.13 | 0.02 | 2.85E-13 | 4.60E-04 | 53.31 |
| rs6742 | 20 | 62374441 | C | 0.16 | 0.03 | 1.30E-07 | 2.41E-04 | 27.87 |
| rs6012503 | 20 | 47251852 | G | 0.09 | 0.02 | 1.62E-07 | 2.37E-04 | 27.44 |
| rs2248461 | 20 | 52792202 | A | -0.11 | 0.02 | 5.33E-10 | 3.33E-04 | 38.55 |
| rs134490 | 22 | 28730175 | T | 0.13 | 0.03 | 6.69E-07 | 2.13E-04 | 24.70 |
| rs9610458 | 22 | 22205353 | T | 0.11 | 0.02 | 4.57E-12 | 4.13E-04 | 47.86 |
| rs140522 | 22 | 50971266 | C | -0.11 | 0.02 | 2.85E-10 | 3.43E-04 | 39.78 |

S2 Table Detailed statistics of selected instrumental variables for multiple sclerosis (β is the estimated effect of the SNP to assess its ability to uniquely predict the outcome; se is the standard error of β; R^2^ is the explanatory power of instrumental variables on exposure factors; F is used to detect the presence of weak instrumental variables)
